# PEI-PEG-Coated Mesoporous Silica Nanoparticles Enhance the Antitumor Activity of Tanshinone IIA and Serve as a Gene Transfer Vector

**DOI:** 10.1155/2021/6756763

**Published:** 2021-11-08

**Authors:** Yinxing Zhu, Miao Yue, Ting Guo, Fang Li, Zhifeng Li, Dazhuang Yang, Mei Lin

**Affiliations:** ^1^Nanjing University of Chinese Medicine, Nanjing, Jiangsu 210023, China; ^2^Institute of Clinical Medicine, Taizhou People's Hospital Affiliated to Nantong University, Taizhou, Jiangsu 225300, China; ^3^Clinical Laboratory, Taizhou People's Hospital Affiliated to Nanjing University of Chinese Medicine, Taizhou, Jiangsu 225300, China

## Abstract

Tanshinone IIA (TanIIA) and gene therapy both hold promising potentials in hepatocellular carcinoma (HCC) treatment. However, low solubility and poor bioavailability of TanIIA limit its clinical application. Similarly, gene therapy with GPC3-shRNA, a type of short hairpin RNAs (shRNAs) capable of silencing the glypican-3 (GPC3) expression, is seriously limited due to its susceptibility to nuclease degradation and high off-target effects. In the present study, polyethyleneimine (PEI)-polyethylene glycol (PEG)-coated mesoporous silica nanoparticles (MSN-PEG) were used as a drug carrier. By encapsulating TanIIA into MSN-PEG, we synthesized MSN-TanIIA-PEG nanoparticles and observed the involved characteristics. This was followed by exploration of antitumor activity on the HepG2 cell lines *in vitro*. Meanwhile, in order to construct GPC3-shRNA plasmids, a shRNA sequence targeting GPC3 was synthesized and cloned into the pSLenti-U6 vector. Accordingly, the performance of MSN-PEG as a gene transfer carrier for GPC3-shRNA gene therapy of HCC *in vitro* was evaluated, including transfection efficiency and DNA binding biological characteristics. The results indicated successful encapsulation of TanIIA in MSN-PEG, which had satisfactory efficacy, favorable dispersity, suitable particle size, and sustained release effect. The *in vitro* anti-HCC effects of nano-TanIIA were greatly improved, which outperformed free-TanIIA in terms of proliferation and invasion inhibition, as well as apoptosis induction of HCC cells. As expected, MSN-PEG possessed excellent gene delivery capacity with good binding, release, and protection from RNase digestion. Using MSN-PEG as a gene carrier, the plasmids were successfully transfected into HepG2 cells, and both the mRNA and protein expressions of GPC3 were significantly downregulated. It was thus concluded that a sustained release TanIIA delivery system for HCC treatment was synthesized and that MSN-PEG could also serve as a gene transfer carrier for gene therapy. More interestingly, MSN-PEG may be a potential delivery platform that combines TanIIA and GPC3-shRNA together to enhance their synergistic effect.

## 1. Introduction

Hepatocellular carcinoma (HCC), as a malignancy characterized by high incidence and mortality, harms people's life and health in a tremendous manner. Seriously, its mortality and morbidity have been steadily increasing over the last few decades [1]. To date, chemotherapy remains the most common treatment for all stages of carcinoma patients. However, several potential chemotherapeutics that can treat HCC still show limitations such as severe adverse reactions and drug resistance [2]. Moreover, the intricacy of the molecular pathogenesis poses great difficulties on seeking cure. Enormous endeavors have thus been made to develop high-efficacy multitarget antineoplastics with less adverse effects.

Several effective plant constituents, which are used in traditional Chinese medicine with insignificant adverse actions, have aroused a wide range of interest as an adjuvant therapy [3]. Among them, TanIIA, an effective component extracted from the *Salvia miltiorrhiza* roots, features high efficacy, natural source, and low toxicity [4]. Based on the existing studies on TanIIA, it exerts a broad spectrum of antitumor activities in a variety of human carcinoma cells by suppressing proliferation and migration, triggering autophagy and apoptosis, and reversing the multidrug resistance [5]. Additionally, TanIIA has a synergistic effect in combination with other chemotherapeutics commonly used in clinics, which makes its application in the cancer and adjuvant therapies promising and offers a new insight into diverse cancer treatments as well [6].

Unsatisfactorily, being a lipophilic constituent, TanIIA is poorly bioavailable, which limits its further application [7]. Due to poor water solubility, it exhibits robust hepatic elimination after oral medication and can be easily eliminated from the circulatory system after intravenous medication [8, 9]. Hence, diverse delivery systems (nanoscale) have been proposed for controlled release of TanIIA, in order to overcome its disadvantages and to elevate its bioavailability [2, 9].

Amongst various nano-based drug delivery platforms developed, mesoporous silica nanoparticles (MSNs) have attracted a considerable attention owing to their good biocompatibility, monodispersity, feeble toxicity, tunable pore size, and large pore volumes, among other characteristics [10]. Despite being in part a potential solution to the foregoing problems with TanIIA, it still cannot escape the influence of the reticuloendothelial system (RES). Rapid elimination by the RES will inevitably hamper the nanosized drugs' absorption efficiency in tumor regions, leading to reduced bioavailability [11]. Being highly hydrophilic and positively charged, PEG is commonly used to decorate nanoparticles [12], which has been proven as one of the most effective methods to improve nanoparticle biodistribution and reduce opsonization by the RES [11].

Studies over the last decades have demonstrated that GPC3 is highly and specifically expressed in HCC, revealing its potential from an encouraging biomarker for the early HCC detection to an effective epitope for targeted HCC treatment. Past several years have witnessed the exploration of the GPC3-targeting gene therapies [13, 14]. As promising as it looks, their applications are severely limited because of the physicochemical traits of nucleotide drugs, including high molecular weight, susceptibility to nuclease degradation, easy missing of target, and anionic charge [15]. This has necessitated carrier design as the gene therapy advances in order to achieve highly efficient drug delivery to the target cells. ShRNAs, the small molecules of RNA, have specific function of gene silencing, which can be delivered to the targets via the support of nanoparticles [16]. Recently, nanostructured carriers such as PEG and PEI or inorganic nanoparticles have shown multiple advantages concerning RNA interference (RNAi) delivery [17]. PEI, as one of the most classic nonviral vectors, is the most broadly applied polycation transfection reagent owing to its high stability and transfection performance, while PEI-25k has been considered the gold standard for nonviral vectors [18, 19]. Moreover, PEI combined with PEG could improve the systemic circulation and prolong the treatment time [19, 20].

**Figure 1:**
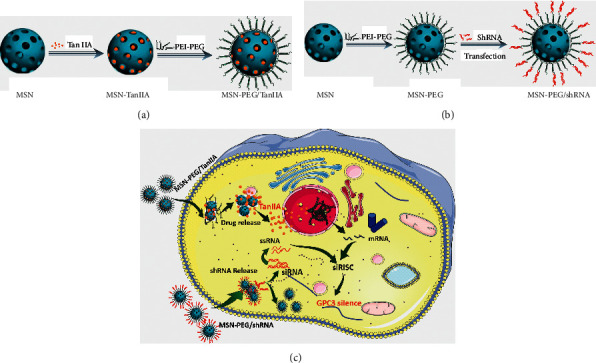
Schematic illustration of the preparation of MSN-PEG nanoparticles and their delivery. (a) Schematic of MSN-TanIIA-PEG preparation. (b) Schematic of MSN-PEG/shRNA preparation. (c) Schematic illustration of the proposed delivery of TanIIA and GPC3-shRNA transfected by MSN-PEG for a synergistic effect *in vitro*. The MSN-TanIIA-PEG and MSN-PEG/GPC3-shRNA nanocomplexes accumulate in the tumor via the EPR effect followed by cellular uptake by endocytosis. TanIIA and GPC3-shRNA are released from the nanocomplexes into the cytoplasm. Then, TanIIA enters into the nucleus, and GPC3-shRNA breaks into siRNAs and siRNA targets to degrade GPC3 mRNA under the assistance of siRISC. Abbreviations: EPR: enhanced permeability and retention, RISC: RNA-induced silencing complex.

Based on the aforementioned theory, this study aims to construct an intelligent nanoplatform to improve the water solubility and bioavailability of TanIIA, which also serves as a vehicle of GPC3-shRNA. Herein, we propose a facile method, where TanIIA was physically adsorbed by mesoporous silica and then surface-modified with PEI-PEG to make it positively charged. In this way, the stability of the complex can be improved, which is conducive to loading GPC3-shRNA plasmids. The physicochemical property elucidation of the complex was accomplished, *in vitro* antitumor activities were investigated, and the feasibility of MSN-PEG as a GPC3-shRNA carrier was explored. Finally, we found that this novel drug delivery system is promising for HCC treatment.  Figure 1 is a schematic illustration of the preparation of MSN-PEG nanoparticles and their delivery.

## 2. Materials and Methods

### 2.1. Materials, Reagents, and Cell Culture

TanIIA (purity ≥ 98% by HPLC) was purchased from Shanghai Yuanye Biotech, Ltd. (Shanghai, China). Monodisperse mesoporous silica nanoparticles (MSNs) were provided by Nanoeast Biotech (Nanjing, China), which are a kind of inorganic nanomaterials with a highly ordered mesoporous structure, good chemical and thermal stability, and a large number of easily modified hydroxyl functional groups on the surface. PEG5k-PEI25k was provided by Tanshui Biotech (Shenzhen, China). The Annexin V-Alexa Fluor 647/PI Apoptosis Assay kit was purchased from FcMACS (Nanjing, China). Lipofectamine 2000, Tango Buffer, and DNase-I enzyme were purchased from Solarbio (Beijing, China). Cell counting kit-8 (CCK-8) was obtained from Beyotime Biotech (Shanghai, China). Fluorescent Hoechst 33342, crystal violet, and the rest of the reagents were purchased from Sangon Biotech (Shanghai, China).

The human HepG2 hepatocellular cancer cell line was obtained from the Shanghai Institute of Cell Research, Chinese Academy of Sciences (Shanghai, China). HepG2 cells were inoculated in Dulbecco's modified Eagle's medium (DMEM) (GIBCO, US) containing 10% fetal bovine serum (GIBCO, US) and supplemented with streptomycin and penicillin (GIBCO, US). The cells were cultured at 37°C in a humidified environment with 5% CO2.

### 2.2. Preparation of the Nanoparticles

The MSN-TanIIA-PEG was prepared according to a film dispersion-ultrasonic method in the published articles [9, 21]. The first step was preparation of TanIIA-loaded MSNs. 5 mg MSN (25 mg/mL) and 500 *μ*g TanIIA (10 mg/mL) were mixed evenly in ethanol, and then 100 *μ*L of water was added slowly into the above solution under ultrasonic condition followed by 2-h incubation in a 37°C shaker. Finally, centrifugation was carried out at 10,000 rpm for 15 min to remove the residual solvent, so as to obtain the required sample (denoted as MSN-TanIIA), which was dried in a 45°C vacuum for 12 h and then stored at 4°C.

The next step was coating of the prepared MSN-TanIIA with PEI-PEG. In a nutshell, 5 mg MSN-TanIIA (10 mg/mL) was dripped into 20 mg (50 mg/mL) PEI-PEG followed by probe sonication for 20 min. After removing extra PEI-PEG and free-TanIIA in the solution via 10-min centrifugation (10,000 rpm), the remaining was washed three times in 2 mL of saline via 5-min centrifugation (10,000 rpm) to get TanIIA-loaded PEI-PEG-coated MSNs, denoted as MSN-TanIIA-PEG. Meanwhile, MSN-PEG was also prepared by the same method.

The entrapped TanIIA in the obtained sample solution was quantified by HPLC. The drug loading capacity (DL, %) and encapsulation efficiency (EE, %) were calculated by the following equations:

(1) $$\begin{array}{c} \text{DL }\left(\%\right)=\left(\frac{\text{amount of TanIIA encapsulated in NPs}}{\text{ total amount of NPs}}\right)\times 100,\\ \text{EE }\left(\%\right)=\left(\frac{\text{amount of TanIIA loaded in NPs}}{\text{initial TanIIA added}}\right)\times 100. \end{array}$$

### 2.3. HPLC Method for Determining TanIIA

#### 2.3.1. Instruments and Conditions

Taking the Chinese Pharmacopoeia (2020 edition) and Liu et al.'s research as reference [22], the high-performance liquid chromatography (HPLC) system (LC-15C; Shimadzu, Japan) with a WondaSil C18 column (4.6 × 250 mm, 5 *μ*m) was used for determination of TanIIA in MSN-TanIIA and MSN-TanIIA-PEG. In addition, the mobile phase of the HPLC system consisted of 70% acetonitrile (A) and 30% ultrapure water (B) at a flow rate of 1 mL/min, column temperature was kept at 30°C, and the detection wavelength was 268 nm.

#### 2.3.2. Standard Solution and Sample Preparation

TanIIA standard solutions (1 mg/mL) were prepared in acetonitrile and stored at −20°C until use. Mobile phase was used to dilute standard solutions to the concentrations of 6.25, 12.5, 25, 50, and 100 *μ*g/mL. Acetonitrile was used to break down the internal structure of the samples, thereby extracting the TanIIA before detecting [23, 24].

### 2.4. Characterization of the Nanoparticles

The physical and chemical properties of MSN-TanIIA-PEG were characterized by transmission electron microscopy (TEM), zeta potential, and dynamic light scattering (DLS). The particle size (nm) and zeta potential (mV) of nanoparticles were evaluated by dynamic light scattering (DLS) at 25°C using the Zeta Plus Zetasizer (Brookhaven Instruments, USA). All the samples were dispersed in deionized water and sonicated before the analysis. The morphology of the uncoated and coated nanoparticles was observed by a JEM-2100 TEM instrument (JEOL, Japan).

### 2.5. *In Vitro* Drug Release

A dialysis technique was employed for the release profile investigation of TanIIA from MSN-TanIIA and MSN-TanIIA-PEG. Initially, 1 mL of the sample solution (1 mg/mL) was added to a dialysis bag (MW:3500), which was soaked in 5 mL of PBS buffer (0.02 M, pH = 7.4, containing 0.2% Tween-80), followed by stirring in a shaking bed at 100 rpm and 37°C away from light. At the preset points of time, each 1.0 mL of sample was removed from the release medium, and then the system was replenished with an equal volume of PBS. The HPLC was finally employed to examine how much TanIIA was released into the release solution.

### 2.6. *In Vitro* Antitumor Activity

To investigate the antitumor activity of MSN-TanIIA-PEG, *in vitro* proliferation, apoptosis, and invasion were assessed (see Sections 2.6.1/2.6.2/2.6.3/2.6.4).

#### 2.6.1. CCK-8 Assay

A CCK-8 assay was conducted to determine the cell proliferation inhibitory effect of the three different TanIIA formulations (free-TanIIA, TanIIA-MSN, and MSN-TanIIA-PEG) on HepG2 cells. In brief, the 3 × 10^4^ cells were seeded into 96-well plates overnight. Then, the cells were intervened with a series of different concentrations (0, 1.25, 2.5, 5, 10, 20, 40, and 80 *μ*g/mL) of free-TanIIA, TanIIA-MSN, and MSN-TanIIA-PEG for 24 h. DMSO was used as a solvent for TanIIA, and thus the HepG2 cells in DMEM containing 0.3% DMSO were used as the untreated control group (0 *μ*g/mL). Moreover, MSN-PEG was also used as a blank nanoparticle control. Subsequently, 10 *µ*L of CCK-8 reagent was added to each well, and the cells were incubated for 30 min at 37°C according to the manufacturer's protocol. Finally, the absorbance was measured using a microplate reader (Thermo Scientific, USA) at a wavelength of 450 nm. The cell growth inhibition rate of the HepG2 cells was calculated according to the formula: cell growth inhibition rate (%) = (1 − experimental absorbance value/control absorbance value) × 100. The 50% inhibitory concentrations (IC_50_) were determined using GraphPad Prism 8.0 software (La Jolla, USA).

#### 2.6.2. Hoechst 33342 Staining Assay

After seeding into 24-well plates overnight, HepG2 cells (1 × 10^5^ cells) were intervened with free-TanIIA, MSN-TanIIA, and MSN-TanIIA-PEG at an equivalent TanIIA concentration of 10 *μ*g/mL. Besides, the concentration of MSN-PEG in the MSN-PEG group was 100 *μ*g/mL. After additional 24-h incubation at 37°C, the cells were washed with cold PBS. Hoechst 33342 (1 *μ*g/mL) was added to each well. After being incubated at 37°C for 15 min, the cells were visualized under a fluorescence microscope (Leica, Germany).

#### 2.6.3. Flow Cytometry Analysis

Before the intervention, the cells were inoculated at 4 × 10^5^ cells per well in 6-well plates overnight. Afterward, the cells were intervened with MSN-PEG (100 *μ*g/mL), free-TanIIA, MSN-TanIIA, and MSN-TanIIA-PEG (at an equivalent TanIIA concentration of 10 *μ*g/mL) for 24 h.

For apoptosis detection, the cells were collected and PBS-washed followed by staining with Annexin V-Alexa Fluor 647 (5 *μ*L) and PI (10 *μ*L, 20 *μ*g/mL) at room temperature protected from light for 15 min and a subsequent resuspension in 500 *μ*L of binding buffer (10 mM HEPES/NaOH, pH 7.4, 140 mM NaCl, 2.5 mM CaCl_2_). The stained cells were detected immediately using FACSCalibur (BD Biosciences, USA).

For the cell cycle assessment, the cells were harvested, PBS-washed, and fixed overnight at 4°C in cold 70% ethanol. After collecting and washing with PBS, the cells were stained using propidium iodide (PI) solution (100 *μ*L; 20 *μ*g/mL PI and 5 *μ*g/mL RNase A in PBS) in the dark for 30 min at ambient temperature followed by FACSCalibur analysis.

#### 2.6.4. Transwell Assay

Cell invasion ability was measured using the transwell assay. In detail, HepG2 cells (4 × 10^5^ cells) were seeded in a 6-well plate and pretreated with MSN-PEG, free-TanIIA, MSN-TanIIA, and MSN-TanIIA-PEG.

Initially, 50 mg/L Matrigel (BD Biosciences, USA) was coated onto the transwell insert membrane at a 1 : 8 dilution at the apical side. After collection from every group, the cells were resuspended in a serum-free medium, and then 1 × 10^5^ cells (200 *μ*L) were transferred into the upper transwell chambers (8 *μ*m; BD Biosciences, USA). The inferior chamber was filled with 500 *μ*L of 10% FBS-containing DMEM. Following 24-h incubation at 37°C, the cells were subjected to twice PBS washing, 30-min fixation in 4% paraformaldehyde, and 20-min staining with crystal violet (0.1%) at room temperature. Then, the cells were washed with water 3 times. After this, a cotton swab was used to remove the nontraveled cells from the upper filter surface in a gentle manner. Finally, the cell pictures were obtained under a microscope and the cell numbers were quantified with ImageJ software. The cell invasion inhibition rate was calculated following this formula: The cell invasion inhibition rate (%) = (cells' count of untreated group − cells' count of the treatment group)/cells' count of untreated group × 100.

### 2.7. Construction and Identification of the GPC3-shRNA Plasmid

The GPC3-shRNA plasmid was constructed as our previous description [25]. The plasmid was purchased from Manfute biotech (Nanjing, China). Human GPC3 sequences (GenBank ID: 2719) were selected as the target site for RNAi. For pSLenti-GPC3-shRNA plasmid construction, driven by the U6 promoter, the annealed oligonucleotides (double-stranded) were inserted into pSLenti plasmids by T4 DNA ligation using AegI-EcoRI restriction sites. The primer sequence (designed by Primer3 software) upstream was 5′-CCGGCCGAAGAAGGGAACTAATTCTCAAGAGAAATTAGTTCCCTTCTTCGGTTTTTTG-3′, and the downstream was 5′-AATTCAAAAAACCGAAGAAGGGAACTAATTTCTCTTGAGAATTAGTTCCCTTCTTCGG-3′. After shaking the bacterial solution, the positive clones were identified by PCR, the reaction conditions of GPC3-shRNA were as follows: initial denaturation at 95°C for 5 min; 30 cycles of 94°C for 30 s, 60°C for 30 s, and extension at 72°C for 60 s, followed by the final extension at 72°C for 10 min. Finally, sequence verification of the resulting pSLenti-U6-GPC3-shRNA plasmids was accomplished via DNA sequencing. Then, the vector NTI software was used to compare and analyze the sequencing results. Meanwhile, to examine the transfection efficiency, GPC3-shRNA-EGFP plasmids were constructed as well.

### 2.8. DNA Binding, Digestion Protection, and Release Experiments

For DNA binding, digestion protection, and release experiments, the methods reported in our previous work were used and the methods description partly reproduced our earlier wording [25].

#### 2.8.1. MSN-PEG Binding with GPC3-shRNA Experiment

To obtain the proper mass ratio of GPC3-shRNA plasmids to MSN-PEG, the same amount of shRNA was incubated with different concentrations of MSN-PEG solutions. GPC3-shRNA plasmids and MSN-PEG were mixed at the mass ratios of 1 : 0 (naked shRNA), 1 : 5, 1 : 10, 1 : 20, 1 : 40, 1 : 60, 1 : 80, and 1 : 100 (final plasmid concentration: 20 ng/*μ*L). After 60 min of incubation at room temperature, 5 *μ*L of each MSN/shRNA complex was subjected to 1% agarose gel electrophoresis (AGE) using 0.5 *μ*g/mL ethidium bromide (EB), with 40-min running of the gels at 110 V with Tris-acetate (TAE) buffer. Retardation of the motion of shRNA was observed with a UV lamp by using a G:BOX Chemi XX9 imaging system (Syngene, UK).

#### 2.8.2. DNA Digestion Protection Capacity

GPC3-shRNA plasmids and MSN-PEG were mixed to form a complex according to a mass ratio of 1 : 20. The complex was then digested with DNase-I enzyme and Tango Buffer at 37°C for 1, 10, 30, 45, and 60 min. Later, the reaction was terminated by adding EDTA solution (100 mmol/L). This was followed by washing, drying of resultants, and a subsequent dissolution in 200 *μ*L ultrapure water. The naked shRNA plasmid DNA (pDNA) was treated the same way and served as a control. Finally, 5 *μ*L of each product was electrophoresed.

#### 2.8.3. DNA Release Capacity of the Complex

GPC3-shRNA plasmids (10 *μ*g) and MSN-PEG were mixed to form a complex according to a mass ratio of 1 : 20. The complex was made to a 500 *μ*L volume by mixing with TE solution. For AGE detection, 5 *μ*L of sample was taken separately at 1, 4, 8, 12, 24, 48, 72, and 96 h.

### 2.9. MSN-PEG Transfection Efficiency Detection

MSN-PEG and GPC3-shRNA-EGFP plasmids were individually dispersed in the DMEM culture medium. MSN-PEG and GPC3-shRNA-EGFP plasmids were incubated at a mass ratio of 20 : 1 for 30 min to make them mix sufficiently.

After HepG2 cells were cultured overnight, the medium was replaced with the above mixture. The culture medium was replaced by fresh DMEM after 5-h incubation. Additional 24-h incubation sustained before harvesting cells. Meanwhile, Lipofectamine 2000 transfection method, which conducted according to the instruction, served as comparison. The two methods' transfection efficiencies were observed using a fluorescence microscope and analyzed via FACSCalibur.

### 2.10. Quantitative Real-Time Polymerase Chain Reaction (PCR) Analysis

Using MSN-PEG as a carrier, the GPC3-shRNA plasmids and negative control were transfected into the HepG2 cells, and 24 h later, cellular mRNA detection of GPC3 was carried out by qRT-PCR. TRIzol reagent (Invitrogen, USA) was used to extract the cellular total RNA as per the instructions of the manufacturer. One microgram of RNA was reverse transcribed into cDNA using ReverTra Ace™ qPCR RT Master Mix (TOYOBO Biotech, Japan). The reaction solution was subjected to 15-min incubation at 37°C and a subsequent 5-min heating to 98°C as per the instructions of the manufacturer. The final step was qRT-PCR analysis on an Applied Biosystems 7500 Real-Time PCR instrument (Thermo Fisher, USA). The comparative Ct (2^−ΔΔCt^) method was used to calculate the relative fold changes of mRNA expression in different groups. The primer sequences are as follows: GPC3 (Beyotime, China): 5′-CCTTTGAAATTGTTCGCCA-3′ (forward) and 5′-CCTGGGTTCATTAGCTGGGTA-3′ (reverse); GAPDH (Beyotime, China): 5′-CATCTTCTTTTGCGTCGCCA-3′ (forward) and 5′-TTAAAAGCAGCCCTGGTGACC-3′ (reverse).

### 2.11. Western Blotting

Total proteins from transfected and untransfected HepG2 cells were extracted using RIPA buffer (Vazyme Biotech, China). BCA assay (Beyotime, China) was conducted to quantify the extracted total proteins. For isolation of the protein samples, 12% sodium dodecyl sulfate polyacrylamide gel electrophoresis (SDS-PAGE) was carried out followed by transfer to polyvinylidene fluoride membranes (0.45 *μ*m) with 300 mA current for 90 min. After transfer, the membranes were blocked in 5% BSA at ambient temperature for 1 h and then incubated overnight using rabbit antibodies against GPC3 (Abcam, USA; diluted 1 : 1000) and a rabbit antibody against GAPDH (Sangon Biotech, China; diluted 1 : 5000) as an internal standard at 4°C to normalize the protein expressions. On the next day, the membranes were further incubated using goat anti-rabbit IgG-HRP (Sangon Biotech, China; diluted 1 : 5000) for 1 h and then subjected to protein visualization using enhanced chemiluminescence (Vazyme Biotech, China). The G:BOX Chemi XX9 imaging system (Syngene, UK) was used for protein visualization.

### 2.12. Statistical Analysis

Quantitative variables were represented as the mean ± standard deviation ($\bar{x}$ ± *s*). *P* < 0.05 was considered statistically significant. The data were determined by one-way ANOVA analysis using GraphPad Prism 8.0 software.

## 3. Results and Discussion

### 3.1. Characterization of Nanoparticles

#### 3.1.1. Particle Size, Zeta Potential, and Morphological Features

It was reported that nanocarriers have high drug loading capacity, excellent tolerability, high stability, and low drug degradation, which achieve controlled release and sustained delivery of antineoplastics [26]. In this study, TanIIA-loaded MSN-PEG nanoparticles were prepared, as described previously. The DL and EE of MSN-TanIIA-PEG nanoparticles were 9.32% and 93.13%, respectively.

**Figure 2:**
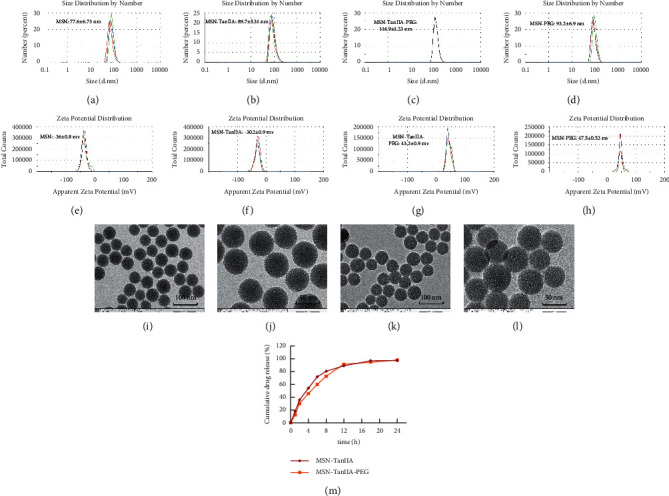
Characterization of nanoparticles. (a–d) The size distribution of nanoparticles. (e–h) The zeta potential of nanoparticles. (i–l) Nanoparticle morphological features were analyzed with TEM at different magnification ratios. (i, j) MSN. (k, l) MSN-TanIIA-PEG. (m) Cumulative drug release curve *in vitro*.

Figures 2(a)–2(h) display the size distribution of the particles, as well as the zeta potential of the MSN, MSN-TanIIA, MSN-TanIIA-PEG, and MSN-PEG. As suggested by the results of DLS assessment, MSN has a hydrodynamic size of 78 nm and a zeta potential of −36.0 mV. With the gradual modification of the MSN nanoparticles with TanIIA and PEI-PEG, the MSN-TanIIA and MSN-TanIIA-PEG exhibited increases in the hydrodynamic size separately to 90 nm and 117 nm. Besides, the surface charges of MSN and MSN-TanIIA were −36 mV and −30 mV, indicating negative charges. Interestingly, both MSN-TanIIA-PEG and MSN-PEG had positive charges with values of 43.2 mV and 47 mV, respectively. This verified the negative-to-positive changes in the surface charges of nanoparticles. The foregoing results imply the successful modification of nanoparticles. Moreover, the positive charges enabled formation of an electrostatic complex by the carrier with shRNA, thereby protecting it from nucleases and facilitating its cellular uptake [27].

TEM analysis was performed to elucidate the morphology of the nanoparticles. According to the TEM micrographs in Figures 2(i)–2(l), the majority of the nanoparticles, regardless of modified or not, was about 50 nm in particle size, which had a regular spherical shape, uniform particle size, and good monodispersity. The surface-modified particles maintained the basic morphology. Additionally, it was obviously observed that the surface mesoporous pores became blurred, and there was a white halo on the nanoparticle periphery, which corresponded to the surface modification with the polymer layer. It has been reported that nanoparticles with a size of 100–200 nm can be quickly eliminated from the blood by macrophages in the RES after entering the circulation. In contrast, the nanoparticles 50–100 nm in size are capable of entering liver cells and target drugs to the liver [2]. According to the EPR effect, nanoparticles with particle size less than 100 nm can easily pass through the interstices of the tumor tissue and thus remain in the tumor tissue [28]. In the present study, the prepared nanoparticle size was about 50 nm, which is in accordance with the preparation requirements of particle size between 50 nm and 100 nm.

#### 3.1.2. *In Vitro* Drug Release

Some studies have demonstrated that the entrapped drug release from the nanoparticles in a sustained manner extended the plasma biological half-life for the natural compounds [29, 30]. For effectiveness validation of the nanoparticles in drug delivery, the *in vitro* release of TanIIA from MSN-TanIIA and MSN-TanIIA-PEG was employed at various time intervals (1, 2, 4, 6, 8, 12, 18, and 24 h), and the curve of TanIIA release was plotted (Figure 2(m)). According to the *in vitro* TanIIA release curve, both MSN-TanIIA and MSN-TanIIA-PEG showed a typical pattern of two-phase release. The TanIIA from MSN-TanIIA and MSN-TanIIA-PEG was initially released at a rapid rate with 71.89% and 59.7% in the first 6 h, which was denoted as incubation period. From 7 to 24 h, the sustained release phase, the cumulative release of the drug gradually increased. After 12 h, the release gradually decreased, which was followed by the smooth release until 24 h. The sustained release reached 97.27% and 97.95% at 24 h for MSN-TanIIA and MSN-TanIIA-PEG, respectively. It was obviously seen that the percentage of TanIIA release in MSN-TanIIA was higher than that of MSN-TanIIA-PEG. The high burst release of MSN-TanIIA may prevent drugs from reaching to the target tissues or cells, thus making it less effective. On the contrary, the sustained release of MSN-TanIIA-PEG was more suitable for the drug release properties of nanoparticles [31].

### 3.2. *In Vitro* Antitumor Activity

#### 3.2.1. Cell Proliferation Inhibition Assay

**Figure 3:**
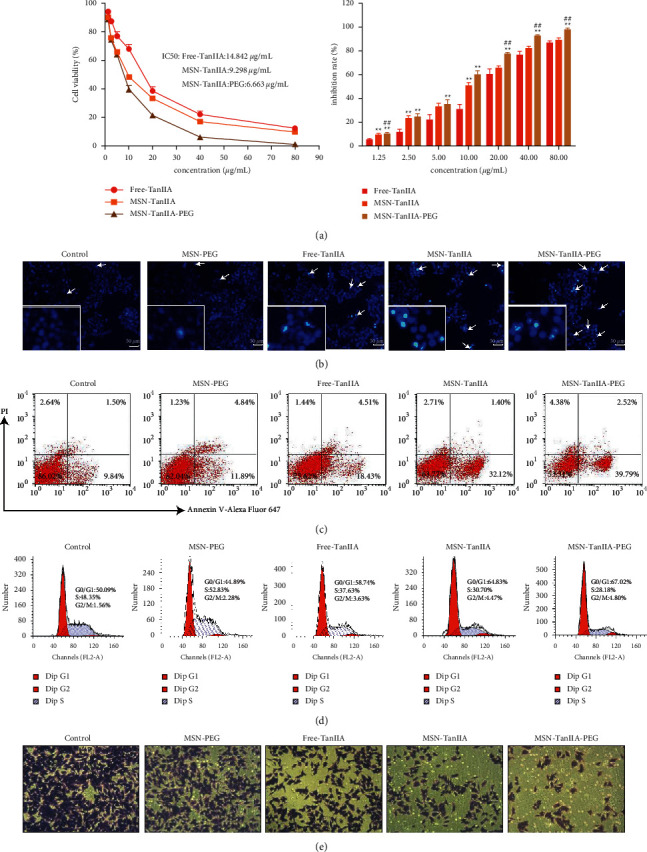
*In vitro* antitumor activity of MSN-TanIIA-PEG. (a) Inhibition rate of HepG2 cells under free-TanIIA, MSN-TanIIA, and MSN-TanIIA-PEG treatment for 24 h (^∗∗^*P* < 0.05 vs. the control group and ^##^*P* < 0.05 vs. other counterparts). (b) Hoechst staining of HepG2 cells incubated with MSN-PEG, free-TanIIA, MSN-TanIIA, and MSN-TanIIA-PEG for 24 h. (c) Apoptosis of HepG2 cells intervened with MSN-PEG, free-TanIIA, MSN-TanIIA, and MSN-TanIIA-PEG for 24 h. (d) Cell cycle distribution of HepG2 cells in different groups. (e) Results of the HepG2 cells' invasion experiment in different experimental groups.

The cell growth inhibition effect was examined under various concentrations of free-TanIIA, MSN-TanIIA, and MSN-TanIIA-PEG (0–80 *μ*g/mL) using the CCK-8 kit. The results revealed increases in the growth inhibition rates of HepG2 cells in a dose-dependent manner. The IC_50_ of free-TanIIA, MSN-TanIIA, and MSN-TanIIA-PEG were 14.842 *μ*g/mL, 9.298 *μ*g/mL, and 6.959 *μ*g/mL, respectively (Figure 3(a)), while the blank nanoparticles (MSN-PEG) and DMSO had no significant effects on the cells' growth. Suggestively, the slower and sustained TanIIA release from the MSN-TanIIA-PEG led to reduced effective dose (IC_50_) and obtained superior therapeutic efficacy *in vitro* to the free-TanIIA for HCC cells. Similar observations have also been reported by Yang et al. [12] and Sun et al. [21], where IC_50_ of many nanoparticles of natural products was lower than that of the free products.

#### 3.2.2. Cell Apoptosis Assays

Hoechst 33342 is typically used for detecting cell apoptosis, and the principle is that it can permeate apoptotic cellular membranes freely, and that apoptotic cells can be identified by the bright blue-stained nuclei that are either condensed or scattered [32]. The apoptotic cell nuclei exhibit a high intensity of fluorescence, while weak fluorescence is noted in the nonapoptotic cells [33]. To evaluate the apoptosis of HepG2 cells induced by MSN-PEG, free-TanIIA, MSN-TanIIA, and MSN-TanIIA-PEG, the cellular nuclei were stained using Hoechst 33342 followed by fluorescence microscope evaluation. Compared with the intact control nuclei, the cells intervened with MSN-PEG showed little difference, while the cells intervened with free-TanIIA, MSN-TanIIA, and MSN-TanIIA-PEG all exhibited nucleus shrinkage and were stained bright blue or showed debris-like lobulation of nuclei (Figure 3(b)). However, within similar sized fields, there were more apoptotic cells in the MSN-TanIIA-PEG group than in the free-TanIIA and MSN-TanIIA groups. This observation is consistent with a previous finding that noisome-coated TanIIA could enhance the apoptosis of HepG2 cells [2].

Subsequently, HepG2 cells intervened with MSN and TanIIA were doubly stained with Annexin V-Alexa Fluor 647 plus PI, and then the cell apoptosis induced by intervention was determined by flow cytometry. As shown in Figure 3(c), the control cells exhibited an exceptionally low apoptosis rate, which was only 8.26 ± 2.83%. In contrast, the cell apoptosis induced by MSN-PEG, free-TanIIA, MSN-TanIIA, and MSN-TanIIA-PEG amounted to 12.81 ± 3.40%, 18.92 ± 3.55%, 30.63 ± 4.09%, and 38.68 ± 6.57%, respectively. Obviously, the cell apoptosis induced by MSN-TanIIA-PEG was far stronger than that induced by other methods (*P* < 0.05). The finding was consistent with the result of Hoechst 33342 staining assay. Moreover, the apoptotic results coincided with the cell proliferation inhibition data as well.

#### 3.2.3. Cell Cycle Assay

Following the verification of cell apoptosis effect induced by MSN-TanIIA-PEG, flow cytometry was utilized to analyze the variation trends of HepG2 cell distribution by the MSN-PEG, free-TanIIA, MSN-TanIIA, and MSN-TanIIA-PEG over the cell cycle phases. According to Figure 3(d), the MSN-TanIIA-PEG intervention group showed an increased *G*0/*G*1 phase cell population compared with the control group, which was also more significant than the MSN-PEG, free-TanIIA, and MSN-TanIIA intervention groups. This finding suggested that nano-TanIIA intervention could affect the arrest of cell cycle at *G*0/*G*1, the phases of DNA replication [34]. In other words, MSN-TanIIA-PEG prevented the duplication of DNA, diminished the S phase cell proportion during DNA synthesis, and inhibited the G2/M phase accumulation of cells. As a result, HepG2 cells' growth and proliferation were effectively suppressed.

This finding is consistent with the previous research showing the ability of TanIIA to arrest cells at the *G*0/*G*1 checkpoint in a dose-related manner [35, 36]. It also agreed with the finding of another study demonstrating a differing effect of the drug-entrapped nanoparticles on the distribution of cell cycle from the free-form drug [37]. However, the effect of TanIIA on the cell cycle distribution remains controversial. Some studies observed that TanIIA could arrest cells at the *S*/*G*2 phases [37, 38]. After HepG2 cells were intervened with MSN-TanIIA-PEG, the *G*0/*G*1 phase cell distribution increased, while the proportion of S and G2/M phase cells decreased, which remains to be further explored.

#### 3.2.4. Cell Invasion Ability Assay

To explore the role of TanIIA in the HCC development, the transwell experiment was employed to examine how TanIIA affected the HepG2 cell invasiveness. Similar to the above outcomes, TanIIA showed remarkable suppression of cell invasion. The cell invasion inhibition rate of the free-TanIIA group was 35.95 ± 8.98%, which was higher than MSN-PEG group's 20.17 ± 8.54% (Figure 3(e)). Compared with the free-TanIIA group, the cell invasion inhibition rate in nano-TanIIA groups (MSN-TanIIA: 59.66 ± 5.09% and MSN-TanIIA-PEG: 71.61 ± 2.58%) was significantly increased. As shown in Figure 3(e), HepG2 cells intervened with MSN-TanIIA-PEG exhibited higher cell invasion inhibition rates than other counterparts. These results indicated that MSN-TanIIA-PEG has the strongest cell invasion inhibition effect on HepG2 cells.

Taken together, our findings suggested that MSN-TanIIA-PEG could suppress HCC cell growth and invasion and promote apoptosis, which may exert a crucial effect on the HCC progression. However, the specific mechanism still needs further verification by molecular biology experiments.

### 3.3. Identification of GPC3-shRNA Plasmid

**Figure 4:**
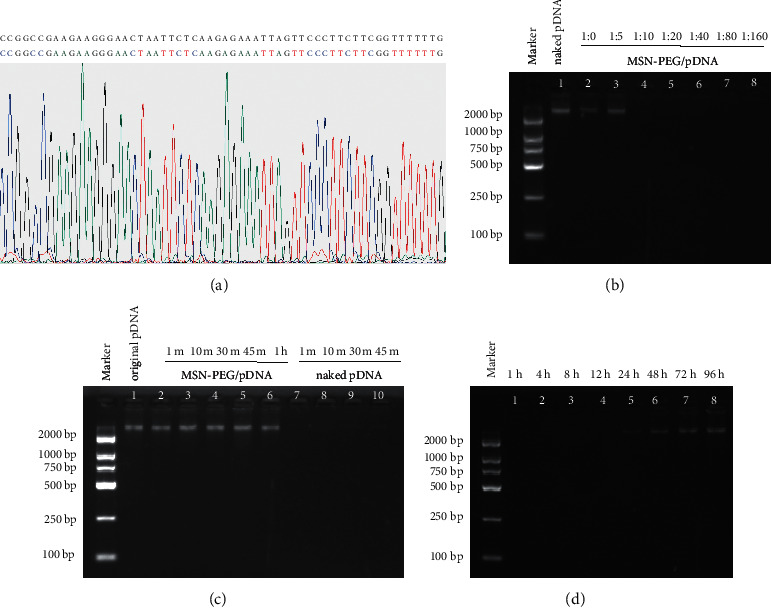
Identification of GPC3-shRNA plasmids and MSN-PEG. (a) GPC3-shRNA sequencing comparison results. (b–d). Binding, digestion, and release of MSN-PEG with GPC3-shRNA plasmid. (b) Electrophoresis image of MSN-PEG with various mass ratios after binding with GPC3-shRNA plasmids. (c) Electrophoresis image of the release experiment of MSN-PEG/GPC3-shRNA complex. (d) Electrophoresis image of the digestion protection experiment of MSN-PEG/GPC3-shRNA complex.

The presence of correct clones was identified by sequencing comparison, which implied that the GPC3-shRNA plasmids were constructed successfully (Figure 4(a)).

### 3.4. Binding, Release, and Digestion of MSN-PEG with GPC3-shRNA Plasmids

Gene therapy targeting GPC3 holds a tremendous potential for HCC treatment, especially the RNAi therapy. The primary difficulty in its clinical application is still the safe and efficient design of delivery carriers. Inspiringly, PEG, a neutral and hydrophilic polymer, has been used widely, which helped lower the cytotoxicity and extend the circulation time. Moreover, there has also been a broad application of positively charged PEI owing to its high DNA condensing and transfection efficiencies [20, 39]. The ability of plasmid binding onto MSN-PEG and the capacities of DNA digestion protection and release were identified by gel retardation assays. As seen from electrophoresis image, when plasmids were added at mass ratios (GPC3-shRNA to MSN-PEG) of 1 : 0, 1 : 5, and 1 : 10, a clear band was observable in the corresponding lane. When the mass ratio was 1 : 20, no band could be observed (Figure 4(b)), indicating that GPC3-shRNA plasmids could be completely loaded onto the nanoparticles, so that the optimal binding ratio was 1 : 20.

Electrophoresis of release assay revealed that no DNA band was observed at 1, 4, 8, 12, or 24 h. Nevertheless, the brightness of the electrophoretic band increased within 48 and 72 h, and there was no significant difference between the 3rd and the 4th day, indicating that MSN-PEG was capable of protecting the pDNA from degradation and reasonably releasing pDNA under appropriate conditions (Figure 4(c)).

In order to observe the stability of the nanoparticle/pDNA complex, the DNase-I digestion experiment was performed. After the complex of MSN-PEG/GPC3-shRNA was added with DNase-I enzyme, the brightness of the electrophoretic band remained stable in the first 1 h. On the contrary, after digestion with DNase-I enzyme, the naked pDNA was almost completely digested within 1 min, and no bands could be seen on the electrophoresis lane, verifying that nanoparticles could effectively protect the pDNA from nuclease digestion (Figure 4(d)).

### 3.5. Evaluation of MSN-PEG Gene Transfection Efficiency

**Figure 5:**
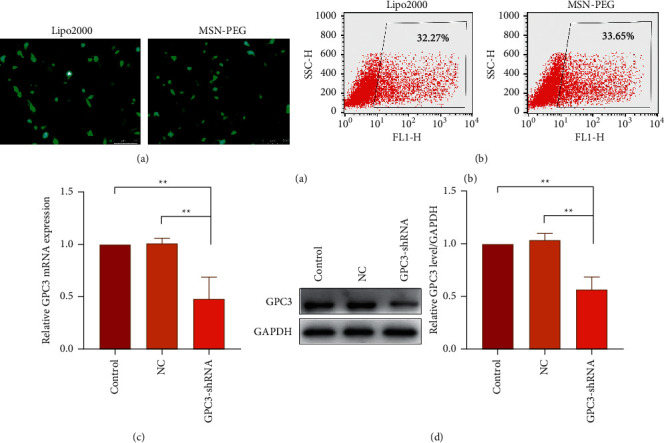
Evaluation of MSN-PEG gene transfection efficiency. (a) The transfection efficiency observed using a fluorescence microscope. (b) The transfection efficiency detected by flow cytometry. (c) GPC3 mRNA expression of transfected HepG2 cells detected by quantitative real-time PCR. (d) GPC3 protein expression of transfected HepG2 cells examined by Western bloting ((a) Protein bands. (b) Quantitative statistical results according to the protein bands). (^∗∗^*P* < 0.05 vs. the transfected with negative control plasmid group and untransfected group).

Using MSN-PEG as a carrier, the GPC3-shRNA-EGFP plasmids were transfected into HepG2 cells for the transfection efficiency observation of GPC3-shRNA plasmids under a fluorescence microscope. As the EGFP expression indicated, no obvious difference was observed in transfection efficiency between MSN-PEG and Lipo2000 in the HepG2 cells (Figure 5(a)). Besides, the transfection efficiency of MSN-PEG was 34.37 ± 1.06% as analyzed by flow cytometry, with no significant difference from the liposome group's 31.78 ± 1.30% (Figure 5(b)). As suggested by the foregoing findings, MSN-PEG has an encouraging potential as a gene-transferring carrier in gene therapy.

HepG2 cells were transfected with GPC3-shRNA via MSN-PEG for 24 h, and then GPC3 gene expression was examined by qRT-PCR combined with Western blotting. It was found that GPC3 mRNA expression in the untransfected (control) group differed indistinctly from that in the negative control (NC) group, although they were both higher than that in the GPC3-shRNA plasmid-transfected group (Figure 5(c)). Correspondingly, the expression level of GPC3 protein was reduced after GPC3-shRNA plasmid transfection, as shown in Figure 5(d). This further confirms the successful establishment of GPC3-shRNA plasmids and the workability of using MSN-PEG to transfer target gene in gene therapy. In a word, it offered a new idea of using MSN-PEG as a vector to carry TanIIA and GPC3-shRNA for comprehensive treatment of HCC in clinics.

## 4. Conclusion

In this study, MSN-TanIIA-PEG with favorable dispersity and biological characteristics was successfully prepared. The antitumor efficacies of nano-TanIIA on HCC *in vitro* were greatly improved, which outperformed free-TanIIA in terms of proliferation and invasion inhibition, and apoptosis induction of HCC cells. Capable of releasing TanIIA into carcinoma cells in a sustained manner, this formulation may be an appropriate candidate for pharmacological application. Nonetheless, the specific mechanism still needs to be further explored, and its efficacy and safety deserve more *in vivo* investigations before clinical trials. Additionally, GPC3-shRNA plasmids were successfully constructed, and MSN-PEG showed an excellent gene transfection efficiency and may thus serve as a carrier for gene therapy. The findings of this study also offer a novel idea for HCC treatment, where MSN-PEG is used as a carrier to combine TanIIA together with GPC3-shRNA.

## Data Availability

The graphics and quantitative data used to support the findings of this study are included within the article.
