# Corrigendum to “PEI-PEG-Coated Mesoporous Silica Nanoparticles Enhance the Antitumor Activity of Tanshinone IIA and Serve as a Gene Transfer Vector”

**DOI:** 10.1155/ecam/9763501

**Published:** 2025-02-26

**Authors:** Yinxing Zhu, Miao Yue, Ting Guo, Fang Li, Zhifeng Li, Dazhuang Yang, Mei Lin

**Affiliations:** ^1^Nanjing University of Chinese Medicine, Nanjing, Jiangsu 210023, China; ^2^Institute of Clinical Medicine, Taizhou People's Hospital Affiliated to Nantong University, Taizhou, Jiangsu 225300, China; ^3^Clinical Laboratory, Taizhou People's Hospital Affiliated to Nanjing University of Chinese Medicine, Taizhou, Jiangsu 225300, China

In the article titled “PEI-PEG-Coated Mesoporous Silica Nanoparticles Enhance the Antitumor Activity of Tanshinone IIA and Serve as a Gene Transfer Vector” [1], there are errors in the mass ratios in Figure 1(b) that should be corrected to 1:5, 1:10, 1:20, 1:40, 1:60, 1:80, and 1:100. In addition, the electrophoresis images in Figures 1(c) and 1(d) are in the wrong order.

The authors explained that these errors were inadvertently introduced during figure assembly. The corrected Figure 1 is as follows.

## Figures and Tables

**Figure 1:**
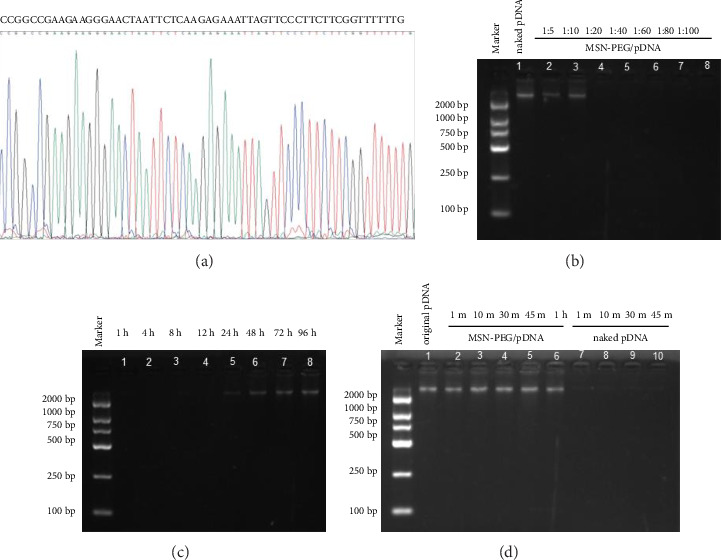
Identification of GPC3-shRNA plasmids and MSN-PEG. (a) GPC3-shRNA sequencing comparison results. (b)–(d) Binding, digestion, and release of MSN-PEG with GPC3-shRNA plasmid. (b) Electrophoresis image of MSN-PEG with various mass ratios after binding with GPC3-shRNA plasmids. (c) Electrophoresis image of the release experiment of MSN-PEG/GPC3-shRNA complex. (d) Electrophoresis image of the digestion protection experiment of MSN-PEG/GPC3-shRNA complex.
